# Hyperprogression of Liver Metastasis With Neoadjuvant Immunotherapy for Soft Tissue Sarcoma

**DOI:** 10.7759/cureus.8575

**Published:** 2020-06-12

**Authors:** Abigail S Chan, Vincent Y Ng, James Snider, Michael E Kallen, Kenneth D Miller

**Affiliations:** 1 Department of Internal Medicine, Sinai Hospital, Baltimore, USA; 2 Department of Orthopedics, University of Maryland Medical Center, Baltimore, USA; 3 Department of Radiation Oncology, Stewart Greenebaum Comprehensive Cancer Center, University of Maryland School of Medicine, Baltimore, USA; 4 Department of Pathology, University of Maryland School of Medicine, Baltimore, USA; 5 Department of Medical Oncology, Stewart Greenebaum Comprehensive Cancer Center, University of Maryland Medical Center, Baltimore, USA; 6 Department of Medical Oncology, Sinai Hospital, Baltimore, USA

**Keywords:** hyperprogression, immunotherapy, soft tissue sarcoma

## Abstract

Hyperprogression associated with immunotherapy has been reported previously with melanoma, non-small cell lung cancer (NSCLC), renal, and urothelial cancers but not with sarcoma. A 63-year old man with a biopsy-proven, localized 13 cm high-grade myxoid/round cell liposarcoma of the thigh was treated with concurrent, neoadjuvant checkpoint inhibitor immunotherapy and radiotherapy. After his subsequent wide surgical resection, he developed small hepatic lesions that rapidly progressed and caused his death, raising the possibility of hyperprogression in this entity.

## Introduction

Soft tissue sarcomas (STS) represent less than 1% of all adult cancers. Due to their rarity and sites of presentation, soft tissue sarcomas are often diagnosed when they are large, increasing their metastatic potential. Radiation and surgical resection comprise the current standard of care for localized disease. The use of chemotherapy remains controversial in this setting due to mixed outcomes in prospective trials. Large studies have shown between 0 to 10% improved survival with chemotherapy, with this approach largely favoring the extremity cohort. Metastatic STS is considered incurable, despite chemotherapy, with an overall poor prognosis [1]. Newer therapies, such as immune checkpoint inhibitors and targeted therapies, are being explored [2]. 

In 1891, Dr. William Coley first described immunotherapy after injecting *Streptococcus pyogenes* and *Serratia marcescens* into inoperable sarcoma patients, hypothesizing that the stimulated immune system would shrink the malignant tumor. Of the 10 sarcoma patients in this series, the majority had a durable response [3]. More than 100 years later, checkpoint inhibitor immunotherapy has been well-described for numerous types of malignancies, including melanoma, bladder carcinoma, renal cell carcinoma, and lung carcinoma [4]. Durable responses with programmed cell death protein 1 (PD-1), programmed death-ligand 1 (PD-L1) and cytotoxic T-lymphocyte-associated protein 4 (CTLA-4) inhibitors have been seen in 10% to 87% of patients across multiple cancer types. The rationale for neoadjuvant immunotherapy for STS includes the low burden of disease, augmentation of radiation-induced abscopal effect, and avoidance of immune-system exhaustion [5-6]. Side effects are comparably minor to traditional cytotoxic chemotherapy [5]. Hyperprogression is rare and only a recently described phenomenon associated with immunotherapy [7]. It has a reported incidence of 9% - 16% based on anecdotal reports in patients with lung cancer, urothelial and renal cancer, and melanoma [4, 7-8]. It must also be recognized that this phenomenon is difficult to distinguish from pseudoprogression until the progression is clearly durable and unrelenting [7]. We, herein, detail a case of possible hyperprogression after treatment with combined PD-L1 and CTLA-4 checkpoint inhibitors, surgery, and radiation in a patient with a soft tissue sarcoma.

## Case presentation

**Figure 1:**
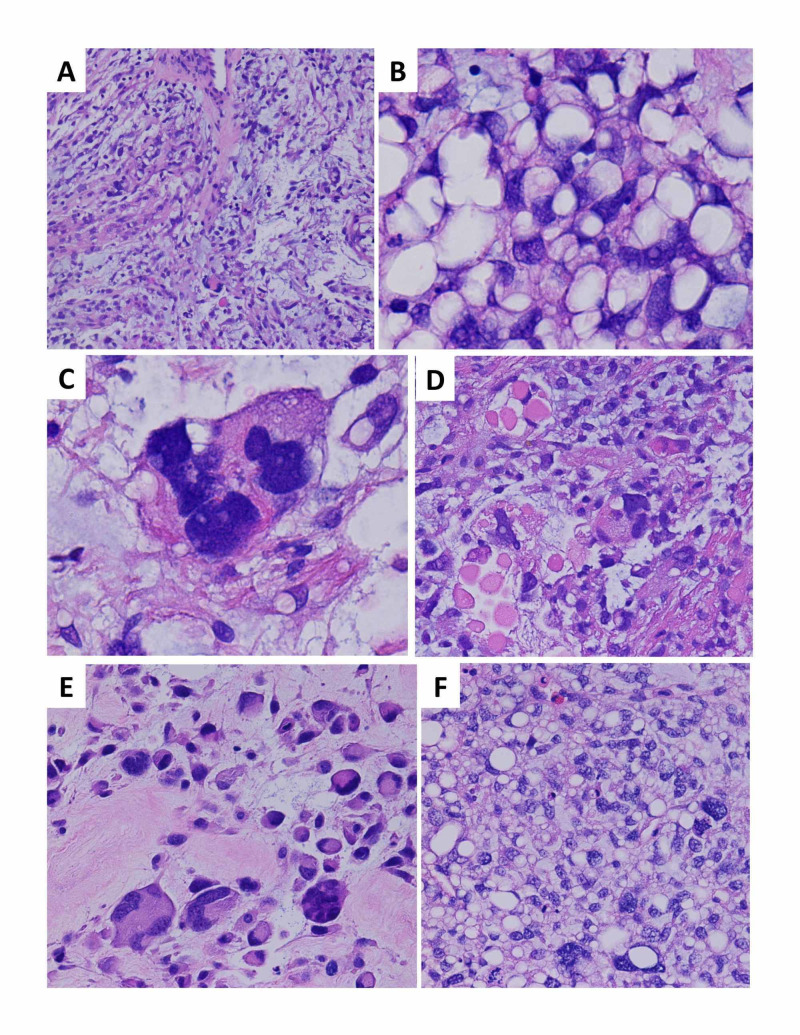
Histologic sections demonstrate a pleomorphic liposarcoma, a high-grade malignancy with aggressive biologic behavior, and morphologic features overlapping with other pleomorphic mesenchymal lesions and liposarcomas (A) The initial core needle biopsy showed areas with a myxoid background, scattered atypical tumor cells, and focal curvilinear vessels, resembling an intermediate grade myxofibrosarcoma, a pattern well-documented to occur in a significant proportion of pleomorphic liposarcomas (H&E stain, 100x magnification); (B) the initial core needle biopsy also demonstrated extensive adipocytic differentiation, which can occur in pleomorphic liposarcomas and obscure the main diagnostic feature, i.e., characteristic lipoblasts with vacuoles crisply indenting the nuclear contours for a “scalloped” appearance.  Lipoblasts are required for the diagnosis of pleomorphic liposarcoma (in contrast to other liposarcoma subtypes in which they are neither necessary nor sufficient).  Lipoblasts can be highly focal and variable in extent, necessitating very thorough tissue sampling and representing a well-known pitfall in misdiagnosis, particularly in small biopsies such as this case (H&E stain, 1000x magnification); (C) extreme pleomorphism and bizarre atypia is a common finding, in contrast to myxoid/round cell liposarcoma (H&E stain, 1000x magnification); (D) intracytoplasmic eosinophilic globules are reported in association with pleomorphic liposarcoma (H&E stain, 400x magnification); (E) the surgical resection demonstrated widespread viable tumor with florid pleomorphism, reflecting neoadjuvant therapy-related effects, and precluding definitive subtyping beyond an unclassifiable pleomorphic sarcoma in the absence of other diagnostic samples from different time points (H&E stain, 1000x magnification); (F) a biopsy of the liver metastasis again showed hypercellularity, prominent adipocytic differentiation, and marked pleomorphism (H&E magnification, 400x magnification) H&E: hematoxylin & eosin

A 63-year-old man with a history of atrial fibrillation presented with a left medial thigh mass in the fall of 2017. He had initially noticed a “lump” a few weeks before seeing his primary care physician. A magnetic resonance imaging (MRI) of the leg showed a large, lobulated, soft tissue mass, measuring 6 x 9.5 x 13 cm, which increased to 9 x 10 x 13 cm a month after his initial visit. Histologic sections from an ultrasound-guided core needle biopsy demonstrated an admixture of hypocellular myxoid and hypercellular adipocytic areas, with frequent large atypical pleomorphic cells scattered throughout both areas, foci of round cell differentiation, and intracytoplasmic eosinophilic inclusions noted, as well as areas of necrosis and high mitotic activity (Figure 1). The characteristic lipoblasts diagnostic of pleomorphic liposarcoma were not identified (a known pitfall in small biopsies), and the diagnosis of a high-grade sarcoma was made with a differential including pleomorphic liposarcoma and high-grade myxofibrosarcoma. Fluorescence in situ hybridization testing was negative for MDM2 amplification and DDIT3 rearrangement, excluding dedifferentiated liposarcoma and myxoid liposarcoma, respectively. The initial positron emission tomography-computed tomography (PET-CT) did not show any metastatic lesions.

**Figure 2:**
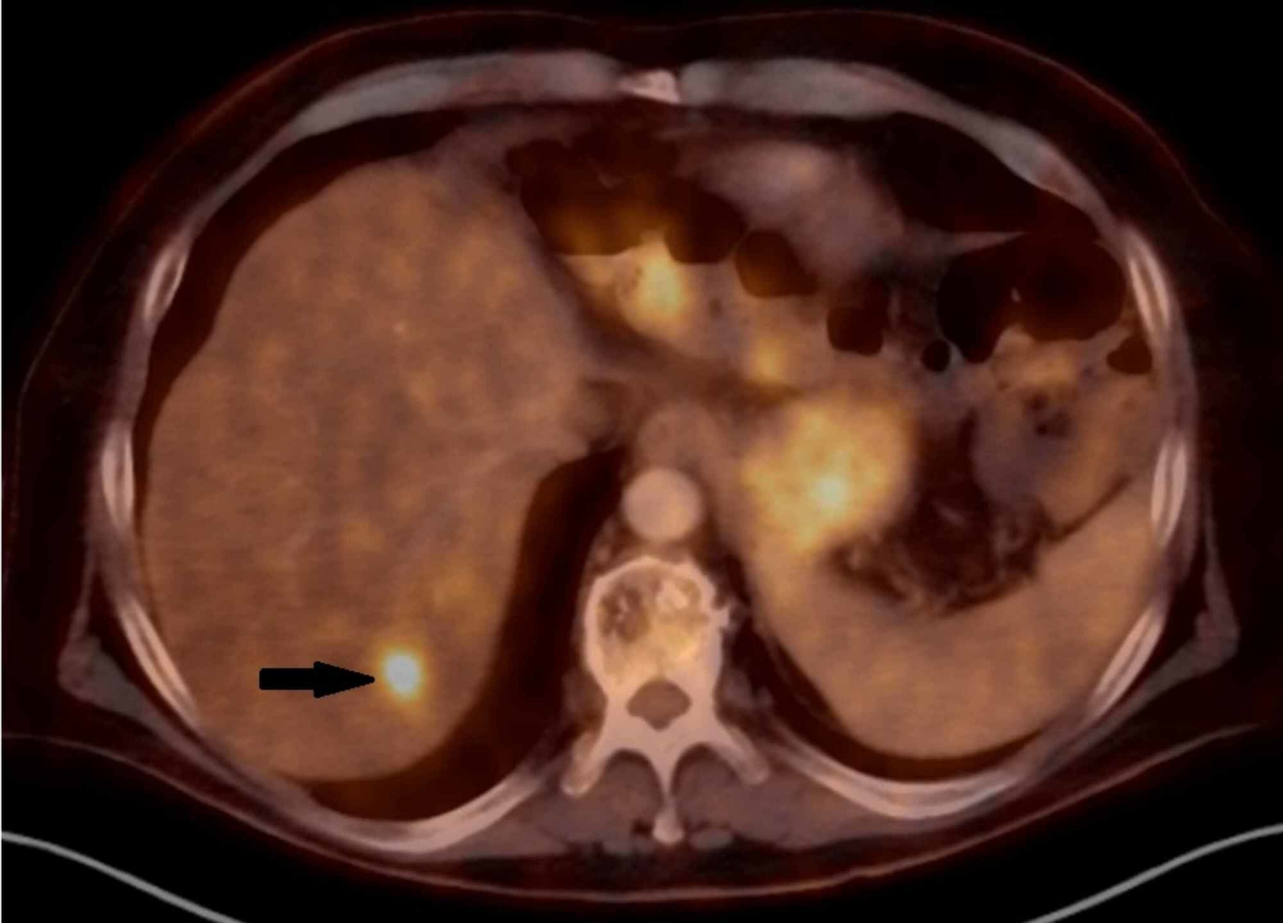
PET-CT revealing a probable hepatic mass (black arrow) PET-CT: positron emission tomography-computed tomography

**Figure 3:**
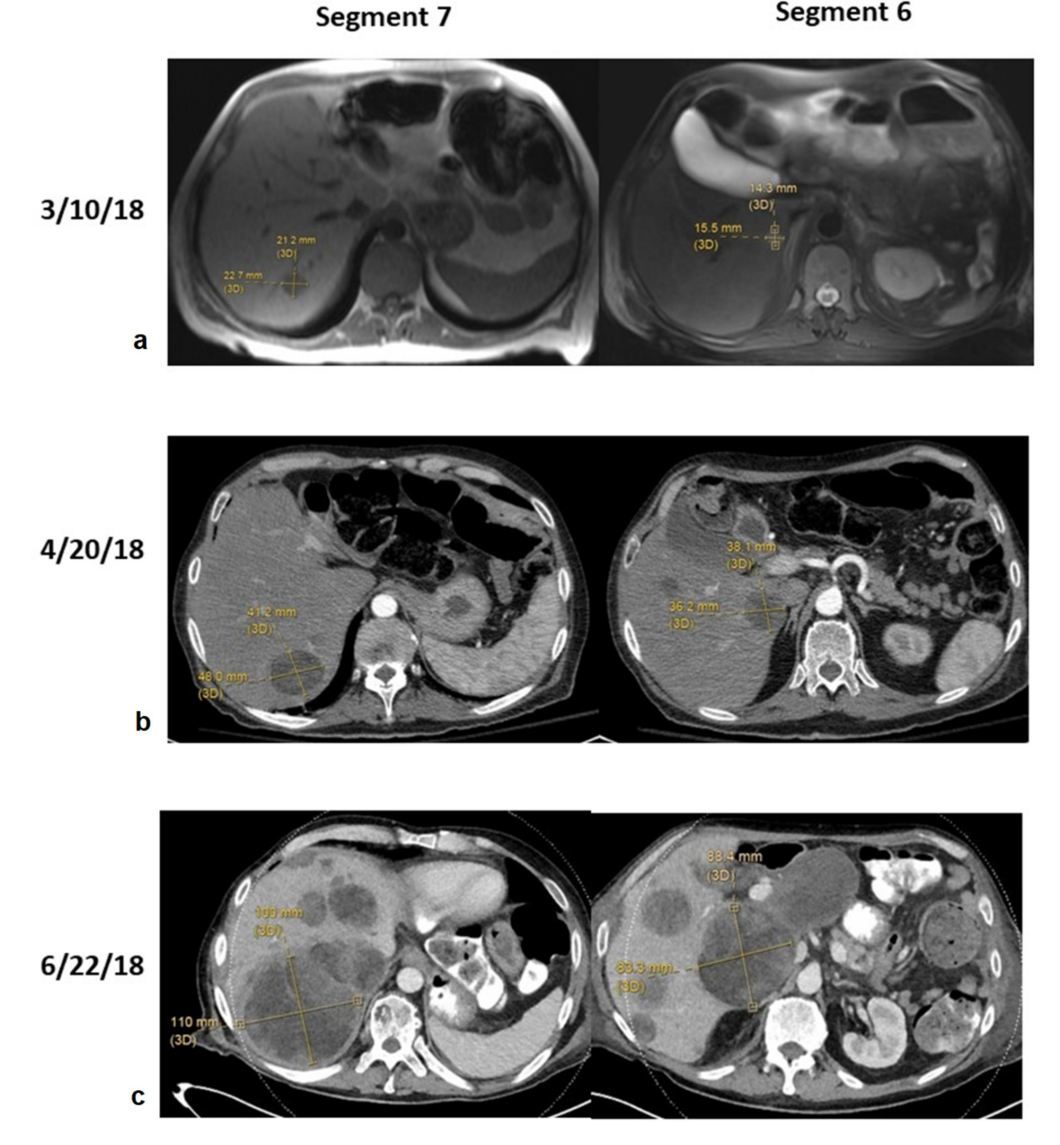
MRI and CT images of segment 6 and 7 at three different time points a) On March 10, 2018, segment 7 was 2.1 x 2.3 x 1.9 cm and segment 6 was 1.5 x 1.4 cm; b) on April 20, 2018, segment 7 was 4.8 x 4.1 cm and segment 6 was 3.8 x 3.6 cm; c) on June 28, 2018, segment 7 was 10.1 x 9.1 x 10 cm and segment 6 was 8.3 x 8.8 cm CT: computed tomography; MRI: magnetic resonance imaging

This patient was included in the institutional NEXIS trial (Nutrition and Exercise in Critical Illness (The NEXIS Trial): A Randomized Trial of Combined Cycle Ergometry and Amino Acids in the ICU, NCT03021902, http://clinicaltrials.gov/ct2/show/NCT03021902), a trial which combines two immunotherapy agents with preoperative radiation with goals of increasing anti-tumor response. He received, per protocol, three doses of IMFINZI® (durvalumab) (1,500 mg) and tremelimumab (75 mg) given once every four weeks with a concurrent single dose, 15 Gy spatially-fractionated radiotherapy (GRID) followed by conventionally fractionated radiotherapy to 50.4 Gy with intensity-modulated radiotherapy (IMRT). He completed immunotherapy and a restaging PET-CT revealed a probable hepatic mass, standardized uptake value (SUV) = 7.1 but without a detectable lesion noted on the CT of the abdomen (Figure 2). Repeat leg magnetic resonance imaging (MRI) revealed a decrease in the size of the mass to 10 x 10 x 11 cm (Table 1). Wide resection of the left thigh mass then followed and demonstrated a 13.5 cm unclassifiable pleomorphic sarcoma with marked treatment effect (60% necrosis), no lymphovascular invasion, and clear margins. Another CT scan of the liver was performed a month later and he was found to have two prominent liver masses (Table 2, Figure 3). Core needle biopsy of the liver lesion demonstrated hypercellular areas with extensive adipocytic differentiation and frequent scattered large atypical forms. The features were consistent with a metastatic high-grade sarcoma from the patient’s primary thigh tumor. Subsequently, serial imaging demonstrated numerous new lesions and very rapid growth of these two liver metastases, with extracapsular extension into the abdominal wall and soft tissues, along with metastatic disease in the left flank soft tissues.

**Table 1:** Sizes of the Left Thigh Mass

	11/1/2017	11/28/17	2/27/18
Left thigh mass	13.2 x 9.5 x 8.6 cm	13 x 10 x 9 cm	10 x 10 x 11 cm

**Table 2:** Prominent Hepatic Masses in Segments 6 and 7 CT: computed tomography; MRI: magnetic resonance imaging

	11/13/17	2/27/18	3/10/18 (MRI)	4/20/18 (CT)	6/22/18 (CT)
Mass in segment 7	Undetectable	Undetectable	2.1 x 2.3 x 1.9 cm	4.8 x 4.1 cm	10.1 x 9.1 x 10 cm
Mass in segment 6	Undetectable	Undetectable	1.5 x 1.4 cm	3.8 x 3.6 cm	8.3 x 8.8 cm

After his surgery, his wounds were slow to heal and required multiple incision and drainage procedures. In addition, he had ongoing problems with anorexia, weight loss, and transaminitis. He decided not to pursue any further systemic therapy and was eventually referred for hospice care.

## Discussion

Soft tissue sarcomas are rare tumors in adults which can develop anywhere, although lower extremities are a common site of origin. A delay in diagnosis is frequently observed due to the presenting symptom of a painless mass. High-risk features, such as a size greater than 5 cm, high-grade tumors, and deep-seated location, warrant immediate biopsy [1]. 

The constellation of histologic features observed in the initial core biopsy are diagnostic of a high-grade sarcoma, with considerations including pleomorphic liposarcoma and high-grade myxofibrosarcoma. Pleomorphic liposarcomas are high-grade sarcomas occurring most often in the limbs of elderly patients and are the least common subtype of liposarcoma. Both the extensive adipocytic differentiation and the hypocellular myxoid areas reminiscent of myxofibrosarcoma are well-described patterns in a significant subset of pleomorphic liposarcoma cases [9]. Severe cytologic atypia and marked pleomorphism are common as well in this entity, though the essential diagnostic lipoblasts can be highly variable in extent and were not present in either of the biopsy samples. This is a notorious pitfall in small biopsies and makes pleomorphic liposarcoma a paradigm of the need for extensive sampling and thorough histologic examination [10]. Pleomorphic liposarcomas have non-distinctive complex karyotypes and are clinically aggressive, with a metastatic rate of 30% - 50% and overall mortality of 40% - 50% [11]. Other diagnostic considerations include a high-grade myxofibrosarcoma, which can demonstrate extensive pseudo-lipoblasts within myxoid areas, although the characteristic curvilinear vessels were not appreciated in this case. Like pleomorphic liposarcomas, myxofibrosarcomas also favor the lower limbs and show nondistinctive and complex karyotypes with no classic recurrent fusions or amplifications. Myxofibrosarcomas are the most common sarcoma of older patients and have a metastatic rate directly linked to grade, with common metastatic sites including the lung, intraabdominal organs (as in this case), and retroperitoneum, and overall survival of approximately 50% [12]. 

The risk of metastasis in STS depends on tumor grade, size, location, and histology. For high-grade STS, the risk of developing metastatic disease is approximately 3% for every 1 cm of tumor diameter [13]. Metastasis to the lungs is the most common pattern found in extremity STS. Hepatic involvement is more frequently present in primary retroperitoneal and visceral STS and is relatively rare in extremity and trunk sarcomas (0.5%) [14-16]. For liver metastases, the options for treatment include the following: complete resection of the liver metastasis when possible, chemotherapy, radiotherapy, or palliative care. Unfortunately, the mortality rate is markedly increased in the presence of liver metastasis. Overall survival has been quoted at seven months [17].

High-grade STS treatment requires a multidisciplinary approach. Neoadjuvant and adjuvant therapy have both been used to improve survival. A meta-analysis of 14 randomized control trials demonstrated a 10% improvement in recurrence-free survival in patients who received adjuvant chemotherapy, but its effect in overall survival was not statistically significant [18]. New approaches have been needed. Somaiah and colleagues conducted a single-center Phase II clinical trial using anti-PD-L1 agent durvalumab in combination with anti-CTLA-4 agent tremelimumab in 49 metastatic sarcoma patients who have undergone several lines of chemotherapy (Poster: Somaiah N, Conley A, Lin H, et al.: A phase II multi-arm study to test the efficacy of durvalumab and tremelimumab in multiple sarcoma subtypes. Connective Tissue Oncology Society Annual Meeting, November 8-11, 2017). Patients received durvalumab, 1,500 mg, and tremelimumab, 75 mg every four weeks for four cycles, followed by durvalumab 1,500 mg every four weeks thereafter. The study concluded that the combination of the two agents showed acceptable tolerance, with a median progression-free survival of four months, and median overall survival of 14.5 months. Clinical trials using other checkpoint inhibitors, including pembrolizumab, Bavencio® (avelumab), and ipilimumab, combined with radiation or chemotherapy agents, are ongoing [2]. Our patient received neoadjuvant combined modality immunotherapy and radiotherapy, followed by surgical resection. Unfortunately, the liver metastases progressed rapidly from undetectable to 10 cm within a three-month period.

The concept of hyperprogression in patients who received immunotherapy was first characterized by Champiat et al. and has been further defined in several ways, including (a) time to treatment failure (TTF) of less than two months, (b) greater than 50% increase in tumor burden compared with pre-immunotherapy imaging, and (c) greater than or equal to a two-fold increase in progression rate [7, 19]. A series of 131 patients at Hospital Gustave Roussy reported an incidence of 9%, with a larger study of 242 patients with non-small cell lung cancer (NSCLC) showing an incidence of 16% [4, 7]. 

Predictive factors of hyperprogression have not been fully defined. However, hyperprogression occurs more often in elderly males with MDM2/MDM4 amplification, epidermal growth factor receptor (EGFR) alterations, low initial tumor volume, and slow-growing disease at baseline. In a case series by Kato et al., four of the six patients (67%) with MDM2 amplification and two out of 10 with EGFR alterations were found to have developed hyperprogression [19]. The histological subtype, the number of previous lines of treatment, and PD-1/PD-L1 expression have not been found to affect its prevalence [8, 19]. Compared with the non-hyperprogressive population, patients who develop this phenomenon have significantly worse overall survival (3.4 months compared to 6.2 months in a study in NSCLC patients) [20]. In contrast, pseudoprogression is an increase in the size of the disease followed by regression. It is confirmed by repeat imaging which should evince eventual stabilization or regression of disease.

Our patient’s clinical course raises concern for hyperprogression based on several unique factors, but it does not meet the exact criteria for hyperprogressive disease (HPD) as the definition does not specifically address the development and growth of new metastases. His hepatic metastases (biopsy-proven) were not evident prior to immunotherapy and developed after he received neoadjuvant radiation and immunotherapy. There was then a dramatic increase in the size of these lesions, approximately doubling every month, excluding pseudoprogression. Other possible explanations would be the presence of a clinically and/or biologically aggressive tumor or the lack of response to immunotherapy. Nevertheless, the rapid growth of his hepatic metastases was unexpected, considering it did not reflect the generally positive results seen in the other STS patients in the NEXIS trial.

## Conclusions

Clinical trials involving immunotherapy in sarcoma treatment have shown some signal of activity, but treatment-related toxicities need to be considered, including the low risk of hyperprogression. Currently, there are no biomarkers, tumoral, or patient factors that can predict this phenomenon. Therefore, frequent serial imaging studies throughout the course of therapy are crucial for monitoring of this unusual complication. Vigilance and clinical experience from a multidisciplinary team, involving the medical and radiation oncologists, radiologists, and surgeons are needed to avoid delayed discontinuation of therapy in the setting of hyperprogression or the early withdrawal of treatment in the case of pseudoprogression. Herein, we have reported a thought-provoking case of potential hyperprogression in a patient with high-grade STS treated with concurrent radiotherapy and dual blockage immunotherapy.

## References

[1] Smolle MA, Andreou D, Tunn PU, Szkandera J, Liegl-Atzwanger B, Leithner A (2017). Diagnosis and treatment of soft-tissue sarcomas of the extremities and trunk. EFORT Open Rev.

[2] Pollack SM, Ingham M, Spraker MB, Schwartz GK (2018). Emerging targeted and immune-based therapies in sarcoma. J Clin Oncol.

[3] McCarthy E (2006). The toxins of William B Coley and the treatment of bone and soft-tissue sarcomas. Iowa Orthop J.

[4] Ferrara R, Caramella C, Texier M (2017). Hyperprogressive disease (HPD) is frequent in non-small cell lung cancer (NSCLC) patients treated with anti PD1/PD-L1 monoclonal antibodies (IO). Ann Oncol.

[5] Seidel JA, Otsuka A, Kabashima K (2018). Anti-PD-1 and anti-CTLA-4 therapies in cancer: mechanisms of action, efficacy, and limitations. Front Oncol.

[6] Keung EZ, Lazar AJ, Torres KE (2018). Phase II study of neoadjuvant checkpoint blockade in patients with surgically resectable undifferentiated pleomorphic sarcoma and dedifferentiated liposarcoma. BMC Cancer.

[7] Champiat S, Dercle L, Ammari S (2017). Hyperprogressive disease (HPD) is a new pattern of progression in cancer patients treated by anti-PD-1/PD-L1. Clin Cancer Res.

[8] Faure M, Rochigneux P, Olive D, Taix S, Brenot-Rossi I, Gilabert M (2018). Hyperprogressive disease in anorectal melanoma treated by PD-1 inhibitors. Front Immunol.

[9] Anderson WJ, Jo VY (2019). Pleomorphic liposarcoma: updates and current differential diagnosis. Semin Diagn Pathol.

[10] Hornick J (2018). Subclassification of pleomorphic sarcomas: how and why should we care?. Ann Diagn Pathol.

[11] Sbaraglia M, Dei Tos AP (2019). Chapter 12: Adipocytic Tumors. Practical Soft Tissue Pathology: A Diagnostic Approach (2nd Edition).

[12] Jo VY, Hornick JL (2019). Chapter 5: Tumors With Myxoid Stroma. Practical Soft Tissue Pathology: A Diagnostic Approach (2nd Edition).

[13] Grimer R (2006). Size matters for sarcomas!. Ann R Coll Surg Engl.

[14] Jaques DP, Coit DG, Casper ES, Brennan MF (1995). Hepatic metastases from soft-tissue sarcoma. Ann Surg.

[15] Asano N, Susa M, Hosaka S (2012). Metastatic patterns of myxoid/round cell liposarcoma: a review of a 25-year experience. Sarcoma.

[16] Estourgie SH, Nielsen GP, Ott MJ (2002). Metastatic patterns of extremity myxoid liposarcoma and their outcome. J Surg Oncol.

[17] Hafner GH, Rao U, Karakousis CP (1995). Liver metastases from soft tissue sarcomas. J Surg Oncol.

[18] Ravi V, Patel S, Benjamin R (2015). Chemotherapy for soft-tissue sarcomas. Oncology (Williston Park).

[19] Kato S, Goodman A, Walavalkar V, Barkauskas D, Sharabi A, Kurzrock R (2017). Hyperprogressors after immunotherapy: analysis of genomic alterations associated with accelerated growth rate. Clin Cancer Res.

[20] Ferrara R, Mezquita L, Texier M (2018). Hyperprogressive disease in patients with advanced non-small cell lung cancer treated with PD-1/PD-L1 inhibitors or with single-agent chemotherapy. JAMA Oncol.

